# Osteocyte-Derived GDF15 Promotes Paclitaxel Resistance, Tumor Growth, and Bone Microenvironment Regulation in Prostate Cancer

**DOI:** 10.3390/cimb48070716

**Published:** 2026-07-14

**Authors:** Weiyi Gao, Meichun Qin, Yi Zhu, Fangming Song, Xin Yang, Wenchu Wang

**Affiliations:** 1 School of Basic Medical Sciences, Guangxi Medical University, Nanning 530021, China; 15772056906@163.com (W.G.); meicqin@163.com (M.Q.); zhuyiqyy@163.com (Y.Z.); 2 Key Laboratory of Longevity and Aging-Related Diseases of Chinese Ministry of Education & Center for Translational Medicine, Guangxi Medical University, Nanning 530021, China; 3 Department of Cell Biology and Genetics, School of Pre-Clinical Medicine, Guangxi Medical University, Nanning 530021, China; 4 Life Sciences Institute, Guangxi Medical University, Nanning 530021, China; songfangming@gxmu.edu.cn; 5 Key Laboratory of Basic Research on Regional Diseases, College of Basic Medical Science, Guangxi Medical University, Nanning 530021, China

**Keywords:** murine osteocyte-like cells, growth differentiation factor 15, prostate cancer, chemoresistance

## Abstract

With changes in lifestyle and dietary patterns, the incidence of prostate cancer in China has been increasing steadily, and it has become one of the most common malignancies in men. Chemotherapy remains a primary treatment for prostate cancer, but the subsequent development of drug resistance by tumor cells markedly compromises its therapeutic efficacy. Growth differentiation factor 15 (GDF15) has been shown to be closely associated with tumor cell proliferation and metastasis; however, its contribution to the drug resistance of prostate cancer cells within the tumor microenvironment has not been systematically investigated. In this study, we treated drug-resistant prostate cancer cells (PC3-TXR and DU145-TXR) with conditioned medium (CM) and examined GDF15 expression by Western blotting, real-time PCR, and ELISA. We then exposed drug-resistant cells to various concentrations of recombinant GDF15 (rGDF15) and CM, and assessed invasive and metastatic abilities as well as sensitivity to paclitaxel using Transwell and CCK-8 assays. We generated GDF15-knockout osteocytes using CRISPR-Cas9 technology and detected the expression of resistance-related signaling pathway components and epithelial–mesenchymal transition (EMT) markers. These findings were further validated through subcutaneous tumor formation assays in mice combined with immunohistochemistry to explore the mechanism by which GDF15 regulates drug resistance and metastasis in the crosstalk between drug-resistant prostate cancer cells and bone cells. Our results revealed that GDF15 expression was significantly upregulated in both drug-resistant prostate cancer cells and their surrounding microenvironment. rGDF15 enhanced the invasion and drug resistance of resistant cells, whereas knockout of GDF15 effectively inhibited these effects. Furthermore, we demonstrated that GDF15 regulates the biological behavior of drug-resistant cells by targeting and modulating the AKT signaling pathway and by promoting EMT initiation and progression. These findings clarify the molecular pathway through which GDF15 governs drug resistance in prostate cancer cells, offering a new direction for the treatment of tumor metastasis and biologically targeted therapy.

## 1. Introduction

Prostate cancer is a common malignancy in men, with a particularly high incidence in Europe and the United States. Among American men, it ranks first in incidence among malignant tumors and is one of the top two causes of cancer-related death [1,2]. In recent years, its incidence has also risen substantially in China, especially in men aged 70 and older, where it has become the most frequent urogenital malignancy [3,4]. The high mortality of prostate cancer is largely attributable to metastasis, and bone is the most common metastatic site; over 80% of patients with advanced disease develop bone metastases [5,6]. Most patients with advanced prostate cancer progress to hormone-independent disease after androgen deprivation therapy. Taxanes are the first-line chemotherapeutic agents for these patients, yet drug resistance and recurrence occur frequently [7], and the intrinsic relationship between hormone independence and chemoresistance remains poorly understood [8,9]. The “seed and soil” theory underscores the crucial role of the bone microenvironment in tumor metastasis [10,11,12], and the bidirectional crosstalk between bone-derived cells and cancer cells can further promote tumor progression and the development of drug resistance [13,14].

Studies on prostate cancer bone metastasis have largely focused on osteoblasts and osteoclasts. In contrast, the regulatory role of osteocytes (OCys), which account for 90–95% of all bone cells, remains poorly defined [14,15]. Osteocytes form an interconnected network that governs bone metabolism and have been shown to influence tumor progression [16,17]; however, their contribution to cancer cell drug resistance is still unclear. Growth differentiation factor 15 (GDF15), a member of the TGF-β superfamily [18,19,20,21,22,23], is closely linked to tumor proliferation, metastasis, and drug resistance [24,25]. We previously demonstrated that prostate cancer cells can induce osteocytes to secrete GDF15, thereby promoting bone metastasis [26]. More recently, we found that drug-resistant prostate cancer cells stimulate osteocytes to produce even greater amounts of GDF15, which in turn enhances epithelial–mesenchymal transition (EMT), invasion, metastasis, and drug resistance of the cancer cells [27,28]. Based on these observations, we hypothesize that during paclitaxel treatment, cancer cells induce osteocytes to secrete GDF15, which then regulates EMT and related signaling pathways to drive drug resistance and metastasis. This study focuses on the regulatory mechanism of GDF15 in the microenvironment formed by drug-resistant prostate cancer cells and osteocytes, aims to delineate the molecular pathways through which GDF15 governs drug resistance and metastasis, and seeks to provide new targets and strategies for clinical therapy.

## 2. Materials and Methods

### 2.1. Cell Line

The human prostate cancer cell lines PC3 and DU145, and their paclitaxel-resistant sublines PC3-TXR and DU145-TXR, were obtained from the Translational Medicine Research Center of Guangxi Medical University, Nanning, China. The mouse osteocyte cell line MLO-Y4 was purchased from Xiamen Yimo Biotechnology Co., Ltd. (Xiamen, China). MLO-Y4-GDF15-KO and MLO-Y4-GDF15-KO-Control cells were generated in this study as described below. PC3-TXR and DU145-TXR cells were cultured in RPMI 1640 medium supplemented with 10% fetal bovine serum (FBS) and 1% penicillin/streptomycin. MLO-Y4, MLO-Y4-GDF15-KO, and MLO-Y4-GDF15-KO-Control cells were cultured in α-MEM containing 5% FBS, 5% bovine calf serum (BCS), and 1% penicillin/streptomycin. All cells were maintained at 37 °C in a humidified atmosphere of 5% CO_2_ and subcultured using trypsinization. Cells were tested for mycoplasma contamination monthly.

### 2.2. Construction of Stable GDF15 Knockout Cell Lines

A lentiviral expressing short guide RNA (sgRNA) targeting the sequence of the GDF15 gene (AACTCAACGCCGACGAGCTA) and negative control (CGCTTCCGCGGCCCGTTCAA) was synthesized and cloned into the GV392 (lentiCRISPRv2) vector with BsmBI sites (purchased from Shanghai Genechem Co., Ltd. (Shanghai, China)), and the recombinant vector was detected by DNA sequencing. The viral vector was transfacted into 293T cells using Lipofectamine 2000 (Invitrogen; Lipofectamine 2000 (Invitrogen, Thermo Fisher Scientific, Inc., Waltham, MA, USA)) together with two helper plasmids, psPAX2 and pMD2.G. Infectious lentiviruses were harvested 72 h post-transfection, subjected to rapid centrifugation to remove cell debris, and then filtered through 0.45 μm cellulose acetate filters. The virus titer was determined by fluorescence-activated cell-sorting analysis of GFP-positive 293T cells and was approximately 1 × 10^9^ transducing units (TUs)/mL medium, and then stored at −80 °C for further use.

### 2.3. Murine Studies

Eighteen 8-week-old male SCID mice (weighing 20–25 g, six mice were used exclusively in preliminary experiments to test the tumorigenicity and growth capacity of the cell lines prior to the formal animal study: statistical analyses were only performed on data from the official experiment, and results of pilot mice were not incorporated) were purchased from Guangdong Vitron Life Sciences Experimental Animal Technology Co., Ltd. (Guangzhou, China). and housed under specific pathogen-free (SPF) conditions at the Experimental Animal Center of Guangxi Medical University. Temperature was maintained at 24–26 °C and relative humidity at 52–62%; drinking water, feed, and bedding were sterilized and bedding was changed twice weekly. All protocols were approved by the Experimental Animal Ethics Review Committee of Guangxi Medical University. Mice were randomly divided into three groups (*n* = 4 per group): (1) DU145-TXR cells alone; (2) DU145-TXR cells plus MLO-Y4-GDF15-KO-Control cells; and (3) DU145-TXR cells plus MLO-Y4-GDF15-KO cells. DU145-TXR cells (1 × 10^7^) alone or mixed with 5 × 10^6^ MLO-Y4-GDF15-KO-Control or MLO-Y4-GDF15-KO cells were resuspended in cold D-PBS, mixed 1:1 (*v*/*v*) with Matrigel, and injected subcutaneously into the right flank in a total volume of 0.12 mL per mouse. After injection, the general condition and tumor formation were monitored three times weekly. Tumor dimensions were measured using calipers, and tumor volume was calculated as V = length × width^2^ × 0.52. At the experimental endpoint, mice were sacrificed, and tumors were excised, fixed, embedded, and sectioned for subsequent analyses. The experimental protocol was approved by the Ethics Committee of Guangxi Medical University, China (approval number: 202512006).

### 2.4. Conditioned Medium

To prepare conditioned medium (CM) from osteocyte lines, MLO-Y4, MLO-Y4-GDF15-KO, and MLO-Y4-GDF15-KO-Control cells were grown to approximately 80% confluence, washed twice with D-PBS, and cultured in RPMI 1640 containing 5% FBS and 1% penicillin/streptomycin for 48 h. Supernatants were collected, centrifuged at 1000× *g* for 3 min to remove cell debris, and stored at −20 °C. Cell counts were determined and CM was normalized by adding medium to a final concentration equivalent to 1 × 10^6^ cells/mL. For treatment of prostate cancer cells, PC3-TXR or DU145-TXR cells at 60% confluence were washed and incubated with the appropriate CM (supplemented with 0.5% FBS for PC3-TXR and 2.5% FBS for DU145-TXR) for 24 or 48 h. Cells and supernatants were then collected for downstream experiments. The preparation procedure of conditioned medium derived from cancer cells was identical to that of MLO-Y4 cell-derived CM, with the only modification that the basal medium was substituted with α-MEM rather than RPMI 1640. Control medium was prepared using the corresponding basal medium supplemented with the same proportion of serum and 1% antibiotic-antimycotic (AA), without extra additives.

### 2.5. ELISA Assays

GDF15 levels in cell culture supernatants were measured using a mouse GDF-15 ELISA kit (Proteintech, Wuhan Sanying Biotechnology, Wuhan, China) according to the manufacturer’s instructions.

### 2.6. Quantitative Real-Time PCR

Total RNA was extracted using TRIzol reagent (TRIzol reagent (Invitrogen, Thermo Fisher Scientific, Waltham, MA, USA)). cDNA was synthesized from 2.0 μg of total RNA using HiScript III RT SuperMix for qPCR (Vazyme, Nanjing, China). Real-time PCR was performed using PowerUp SYBR Green Master Mix (Applied Biosystems, Foster City, CA, USA) on a 7300 Real-Time PCR System (Applied Biosystems, Foster City, CA, USA) in a 15 μL reaction volume. Cycling conditions were as follows: 95 °C for 30 s, followed by 40 cycles of 95 °C for 30 s and 65 °C for 20 s. Primers for human GDF15 (catalog no. 3300398119, 3300398120) and mouse GDF15 (catalog no. 3300832110, 3300832111) were purchased from Sangon Biotech. GAPDH was used as the internal reference. All reactions were performed in triplicate, and experiments were independently repeated three times. Relative expression was calculated using the 2^−ΔΔCt^ method.

### 2.7. Generation of Gene Knockout Cells

To establish stable GDF15-knockout MLO-Y4 cells, MLO-Y4 cells were seeded in 12-well plates, cultured overnight, and transduced with Cas9 lentiviral particles (catalog no. GCEL5001199, GeneChem Co., Ltd., Shanghai, China). At 36 h post-transduction, stably transduced cells were selected using 5.5 μg/mL puromycin, yielding MLO-Y4-GDF15-KO and the empty vector control line MLO-Y4-GDF15-KO-Control.

### 2.8. Transwell Migration and Matrigel Invasion Assays

Migration and invasion were assessed using Transwell chambers (Corning Inc., Corning, NY, USA). For invasion assays, inserts were pre-coated with Matrigel; for migration assays, uncoated inserts were used. PC3-TXR (1–2 × 10^5^ cells) or DU145-TXR (0.5–1 × 10^5^ cells) were suspended in 200 μL serum-free RPMI 1640 and seeded into the 700 μL upper chamber. The lower chamber was filled with control medium, CM, or recombinant GDF15 (Cat. 8944-GD-025, Cat. 8944-GD-025, R&D Systems, Minneapolis, MN, USA). All experimental and control groups were additionally supplemented with FBS to a final concentration of 20%. The medium for the blank control group consisted of RPMI 1640 medium supplemented with 20% FBS and 1% antibiotic-antimycotic (AA). After incubation, cells that failed to migrate or invade were gently wiped off from the upper surface of the inserts with sterile cotton swabs, followed by fixation, staining, and microscopic quantification. After incubation, cells on the lower surface of the filter were stained with crystal violet solution (Jizhi Biotechnology, Shanghai, China), photographed under a light microscope at 200× magnification, and counted in five random fields per insert. Each condition was assayed in triplicate.

### 2.9. CCK-8 Assays

PC3-TXR (4000 cells/well) or DU145-TXR (5000 cells/well) were seeded in 96-well plates and cultured for 24 h before treatment with rGDF15, CM, control medium, or the AKT inhibitor MK2206 for the indicated times. At each time point, 10 μL of CCK-8 reagent (Dojindo Laboratories, Kumamoto, Japan) was added to 100 μL of culture medium and incubated at 37 °C for 4 h. Absorbance was measured at 450 nm using a microplate reader (Thermo Fisher Scientific).

### 2.10. Western Blotting Assays

Cells were lysed in RIPA buffer containing protease and phosphatase inhibitors. Protein concentrations were determined by BCA assay (Beyotime Biotechnology, Shanghai, China). Equal amounts of protein (50 μg) were separated by 10% SDS-PAGE and transferred onto PVDF membranes. Membranes were blocked with 5% non-fat milk in TBST (0.1% Tween-20) for 1.5 h at room temperature and incubated overnight at 4 °C with primary antibodies. The following primary antibodies were used: GDF15 (CST 9479S), Notch1 (CST 3608T), E-cadherin (Proteintech 20874-1-AP), N-cadherin (Proteintech 82968-1-RR), β-catenin (Proteintech 51067-2-AP), Twist1 (Proteintech 25465-1-AP), and Claudin-1 (Proteintech 28674-1-AP). All antibodies were diluted according to the manufacturers’ recommendations. After washing, membranes were incubated with HRP-conjugated secondary antibodies (Invitrogen 31460) and signals were detected by enhanced chemiluminescence.

### 2.11. Scratch Wound Healing Assay

Parallel reference lines were drawn at the bottom of 6-well plates in advance. PC3-TXR and DU145-TXR cells treated with different conditioned media were seeded and cultured until reaching 90% confluence. Uniform scratches were created vertically to the marker lines using combined pipette tips. Cell debris was washed away with D-PBS, and serum-free medium was added to exclude the interference of cell proliferation. Images of scratch wounds at fixed positions were captured at 0, 12, and 24 h under a 10× objective lens. ImageJ 1.53e software was used to quantify the wound closure area, and GraphPad Prism 8.0 was utilized for statistical analysis and graph plotting.

### 2.12. Statistical Analysis

All data were analyzed using GraphPad Prism 8.0 and are presented as mean ± SD. Comparisons between two groups were made using unpaired Student’s *t*-test; comparisons among multiple groups were performed by one-way ANOVA. When the assumption of homogeneity of variance was not met, non-parametric tests were applied. A *p* value < 0.05 was considered statistically significant (* *p* < 0.05, ** *p* < 0.01, *** *p* < 0.001).

## 3. Results

### 3.1. Drug-Resistant Prostate Cancer Cells Secrete High Levels of GDF15, Which Enhances Their Migration, Invasion, and Resistance to Paclitaxel

**Figure 1:**
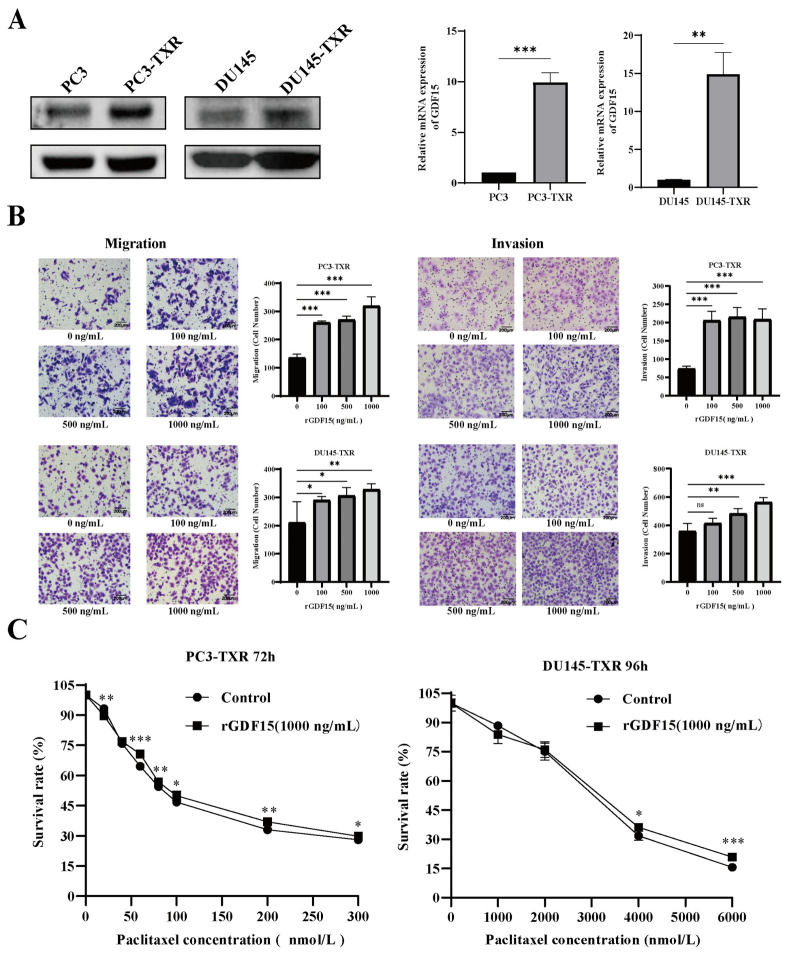
Drug-resistant prostate cancer cells exhibit elevated GDF15 expression and rGDF15 enhances their migration, invasion, and paclitaxel resistance. (**A**) GDF15 protein and mRNA levels in parental PC3 and DU145 cells and drug-resistant PC3-TXR and DU145-TXR cells were assessed by Western blotting and quantitative real-time PCR. (**B**) Transwell assays were used to evaluate the migratory and invasive capacities of PC3-TXR and DU145-TXR cells treated with rGDF15 at 0, 100, 500, and 1000 ng/mL. (**C**) Paclitaxel sensitivity was determined by CCK-8 assay; rGDF15 treatment significantly enhanced paclitaxel resistance in PC3-TXR cells at 72 h and in DU145-TXR cells at 96 h compared with the control group. Data are shown as the mean ± SD of three independent experiments. * *p* < 0.05, ** *p* < 0.01, *** *p* < 0.001; ns, not significant.

To determine whether GDF15 expression differs between parental and drug-resistant prostate cancer cells, we first compared GDF15 levels in PC3-TXR and DU145-TXR cells with their parental counterparts. Both GDF15 mRNA and protein were markedly elevated in PC3-TXR and DU145-TXR cells relative to parental PC3 and DU145 cells (Figure 1A). To assess whether GDF15 influences migration, invasion, and paclitaxel sensitivity of drug-resistant cells, PC3-TXR and DU145-TXR cells were treated with recombinant GDF15 (rGDF15). rGDF15 significantly enhanced the migratory and invasive capacities of both resistant lines, and each line displayed a dose-dependent response (Figure 1B). To further evaluate the effect of GDF15 on paclitaxel resistance, IC50 values were determined. The IC50 of PC3-TXR control cells was 110.67 ± 7.57 nmol/L, whereas the IC50 of PC3-TXR cells treated with rGDF15 was 122.97 ± 8.44 nmol/L; similarly, the IC50 of DU145-TXR control cells was 2555.33 ± 374.68 nmol/L compared with 3438.67 ± 281.41 nmol/L for the rGDF15-treated group (*p* < 0.05) (Figure 1C). Taken together, these results demonstrate that drug-resistant prostate cancer cells secrete high levels of GDF15, and that GDF15 enhances their migration, invasion, and resistance to paclitaxel.

### 3.2. Drug-Resistant Prostate Cancer Cells Interact with MLO-Y4 Osteocytes to Upregulate GDF15 Secretion, Which Enhances Their Migration, Invasion, and Paclitaxel Resistance

**Figure 2:**
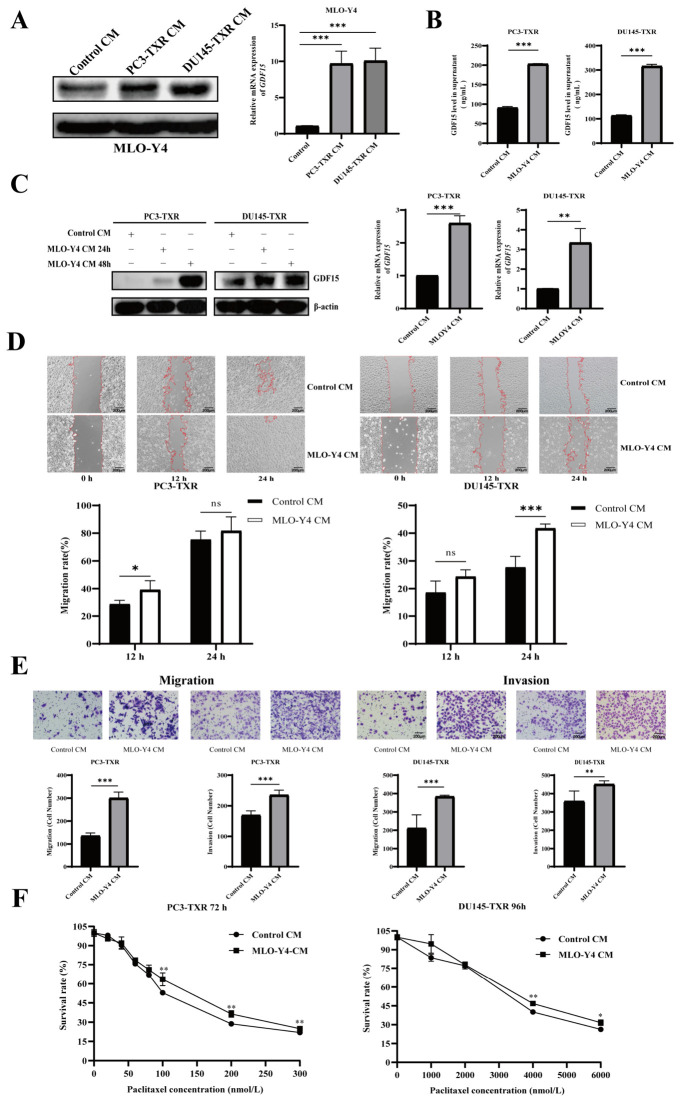
GDF15 secreted by MLO-Y4 osteocytes and prostate cancer cells into the microenvironment upregulates GDF15 expression in both cell types. (**A**) MLO-Y4 cells were treated with conditioned media (CM) from paclitaxel-resistant PC3-TXR and DU145-TXR prostate cancer cell lines or control CM for 24 h. Total cell lysates and RNA were collected and subjected to immunoblotting for GDF15 protein and real-time PCR for GDF15 mRNA, respectively. (**B**) PC3-TXR and DU145-TXR cells were treated with MLO-Y4 CM or control CM. Supernatants were collected and analyzed by ELISA for secreted GDF15. (**C**) PC3-TXR and DU145-TXR cells were treated with MLO-Y4 CM or control CM. After 24 and 48 h, cell lysates were subjected to immunoblotting for GDF15 protein. Total RNA was subjected to real-time PCR for GDF15 mRNA. (**D**) Migratory capacity of PC3-TXR and DU145-TXR cells was assessed by wound healing assay following treatment with MLO-Y4 CM or control CM. Wounded monolayers were photographed at 0, 12, and 24 h; wound widths were measured and the repair rate was calculated. (**E**) Migratory and invasive abilities of PC3-TXR and DU145-TXR cells were evaluated by Transwell assay after treatment with MLO-Y4 CM or control CM. Cells were counted in five random microscopic fields per filter (200× magnification). (**F**) CCK-8 assay showed that MLO-Y4 CM significantly enhanced paclitaxel resistance in PC3-TXR cells at 72 h and DU145-TXR cells at 96 h compared with control CM. Data are shown as the mean ± SEM of three independent experiments. * *p* < 0.05, ** *p* < 0.01, *** *p* < 0.001.

To determine if bone cells and drug-resistant prostate cancer cells crosstalk to elevate GDF15 in the microenvironment, MLO-Y4 osteocytes were treated with CM from PC3-TXR and DU145-TXR cells. After 48 h, total protein and RNA were harvested from MLO-Y4 cells for Western blotting and qPCR analysis. CM from both drug-resistant lines induced elevated GDF15 protein and mRNA expression in MLO-Y4 cells (Figure 2A).

Having established that PC3-TXR and DU145-TXR CM upregulate GDF15 in MLO-Y4 cells, we next examined whether MLO-Y4 cells could reciprocally induce GDF15 expression in drug-resistant prostate cancer cells and contribute to GDF15 levels in the microenvironment. The two drug-resistant lines were treated with MLO-Y4 CM for 48 h, after which culture supernatants, whole-cell protein, and RNA were collected for ELISA, Western blotting, and qPCR, respectively. Compared with the control group, MLO-Y4 CM induced elevated GDF15 in the supernatants as well as increased GDF15 protein and mRNA levels in both resistant lines (Figure 2B,C). These findings indicate that drug-resistant prostate cancer cells and MLO-Y4 osteocytes mutually promote GDF15 secretion in the microenvironment.

To determine whether the GDF15 secreted by MLO-Y4 cells into the microenvironment could recapitulate the ability of recombinant GDF15 to enhance migration, invasion, and paclitaxel resistance of drug-resistant prostate cancer cells, we evaluated the effect of MLO-Y4 CM on these properties. Treatment with MLO-Y4 CM promoted the migration and invasion of PC3-TXR and DU145-TXR cells (Figure 2D,E), consistent with the effect observed following the addition of rGDF15.

To further assess whether microenvironment-derived GDF15 affects paclitaxel sensitivity similarly to recombinant GDF15, CCK-8 assays were performed (Figure 2F). The IC50 of PC3-TXR control cells (96 h) was 146.7 ± 4.04 nmol/L, compared with 155.57 ± 7.4 nmol/L for PC3-TXR cells treated with MLO-Y4 CM; the IC50 of DU145-TXR control cells (96 h) was 3148.33 ± 242.03 nmol/L, compared with 4194.33 ± 409.14 nmol/L for DU145-TXR cells exposed to MLO-Y4 CM. These results demonstrate that, in the bone microenvironment, osteocyte-derived factors induce enhanced migration, invasion, and paclitaxel resistance of drug-resistant prostate cancer cells.

### 3.3. GDF15 Promotes Prostate Cancer Migration, Invasion, and Metastasis and Reduces Paclitaxel Sensitivity in the Microenvironment

**Figure 3:**
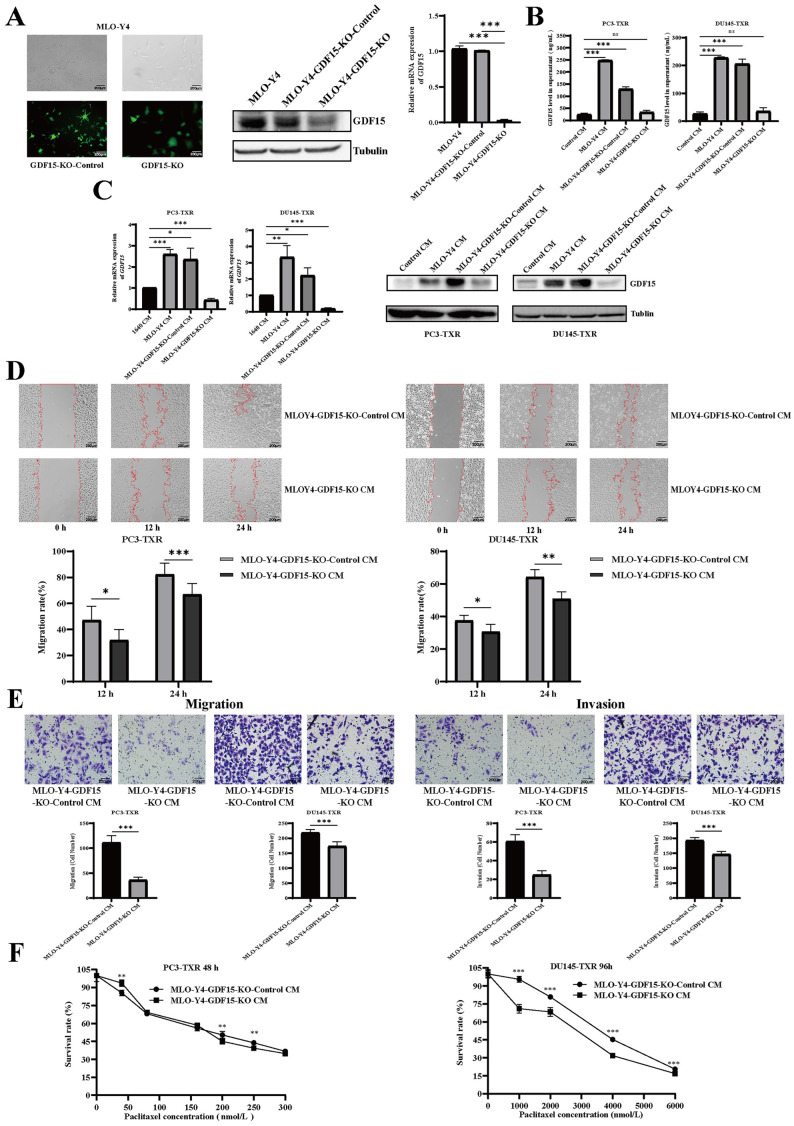
GDF15 knockout in MLO-Y4 osteocytes reduces GDF15 expression and attenuates migration, invasion, and paclitaxel resistance in drug-resistant prostate cancer cells. (**A**) Validation of GDF15 knockout MLO-Y4 cell lines. Fluorescence microscopy (10× objective) showed that the majority of cells were fluorescence positive. Total protein was extracted from parental, GDF15-KO-Control, and GDF15-KO MLO-Y4 cells and processed via Western blotting to detect endogenous GDF15 protein levels. Total RNA isolated from the three MLO-Y4 cell groups was subjected to q-PCR to measure GDF15 mRNA expression. (**B**) PC3-TXR and DU145-TXR cells were treated with control CM, MLO-Y4 CM, MLO-Y4-GDF15-KO-Control CM, or MLO-Y4-GDF15-KO CM for 24 h. Cell lysates and RNA were collected and subjected to Western blotting and real-time PCR for GDF15, respectively. (**C**) PC3-TXR and DU145-TXR cells were treated with control CM, MLO-Y4 CM, MLO-Y4-GDF15-KO-Control CM, or MLO-Y4-GDF15-KO CM for 24 h. Culture supernatants were collected and subjected to ELISA to quantify secreted GDF15. (**D**) Wound healing assays was used to assess the migratory ability of PC3-TXR and DU145-TXR cells treated with control CM, MLO-Y4 CM, MLO-Y4-GDF15-KO-Control CM, or MLO-Y4-GDF15-KO CM. Wounded monolayers were photographed at 0, 12, and 24 h; wound widths were measured (three sites per group) and the repair rate was calculated. (**E**) Transwell assays were performed to evaluate the migratory and invasive abilities of PC3-TXR and DU145-TXR cells after treatment with the indicated CM. Cells were counted in five random microscopic fields per filter (200× magnification). (**F**) CCK-8 assays were performed to evaluate paclitaxel sensitivity of PC3-TXR and DU145-TXR cells cultured with MLO-Y4-GDF15-KO-Control CM or MLO-Y4-GDF15-KO CM. Cell viability was detected at 48 h for PC3-TXR cells and 96 h for DU145-TXR cells for subsequent IC50 calculation. Data are shown as the mean ± SEM of three independent experiments. * *p* < 0.05, ** *p* < 0.01, *** *p* < 0.001; ns, not significant.

To explore whether the ability of bone cells to promote the metastasis, migration, and invasion of drug-resistant prostate cancer cells and their sensitivity to paclitaxel depend on GDF15, in this study, GDF15-knockout MLO-Y4-GDF15-KO cells and the control group MLO-Y4-GDF15-KO-Control control cells were used (Figure 3A). Fluorescence images were captured under a 10× objective lens of the fluorescence microscope. The transduced cells expressed green fluorescent protein (EGFP), so successfully transduced cells exhibited distinct green fluorescence signals, which were used to evaluate the lentiviral transduction efficiency. The conditioned medium was collected respectively for the treatment of PC3-TXR and DU145-TXR prostate cancer drug-resistant cells; after treatment, the cell supernatants, total proteins, and RNA were collected to detect whether the induction effect of osteocytes on drug-resistant prostate cancer cells changed. The results showed that the conditioned medium of MLO-Y4-GDF15-KO cells (MLO-Y4-GDF15-KO CM) could significantly reduce the high expression of GDF15 in drug-resistant prostate cancer cells induced by bone cell-derived GDF15 (Figure 3B,C). At the same time, MLO-Y4-GDF15-KO CM significantly weakened the enhancing effect of bone cells on the migration, invasion, and metastasis abilities of drug-resistant prostate cancer cells (Figure 3D,E), as well as the enhancing effect on their paclitaxel resistance (Figure 3F). The results of the CCK-8 assays showed that the IC50 of PC3-TXR cells (48 h) treated with MLO-Y4-GDF15-KO-Control conditioned medium was 171.87 ± 17.44 nmol/L; the IC50 of PC3-TXR cells (48 h) treated with MLO-Y4-GDF15-KO conditioned medium was 149.3 ± 29.25 nmol/L; the IC50 of DU145-TXR cells (96 h) treated with MLO-Y4-GDF15-KO-Control conditioned medium was 3426 ± 214.18 nmol/L; and the IC50 of DU145-TXR cells (96 h) treated with MLO-Y4-GDF15-KO conditioned medium was 2315.67 ± 175.32 nmol/L. These results indicate that in the bone tumor microenvironment, GDF15 secreted by bone cells into the microenvironment can significantly enhance the metastasis, migration, and invasion abilities of prostate cancer-resistant cells, and further enhance their resistance to paclitaxel.

### 3.4. GDF15 Upregulates AKT1/2/3 Expression in Drug-Resistant Prostate Cancer Cells

To identify downstream targets of osteocyte-derived GDF15 in drug-resistant prostate cancer cells, PC3-TXR and DU145-TXR cells were treated with control CM or MLO-Y4 CM, and changes in transcription factor expression were evaluated. Compared with control CM, MLO-Y4 CM altered the expression of multiple transcription factors in both drug-resistant cell lines [29,30,31,32,33,34,35,36,37]. Notably, MLO-Y4-derived GDF15 consistently upregulated the expression of AKT1/2/3 in both PC3-TXR and DU145-TXR cells, and this upregulation increased with elevated GDF15 concentrations in the microenvironment, indicating a consistent effect across drug-resistant prostate cancer cells.

**Figure 4:**
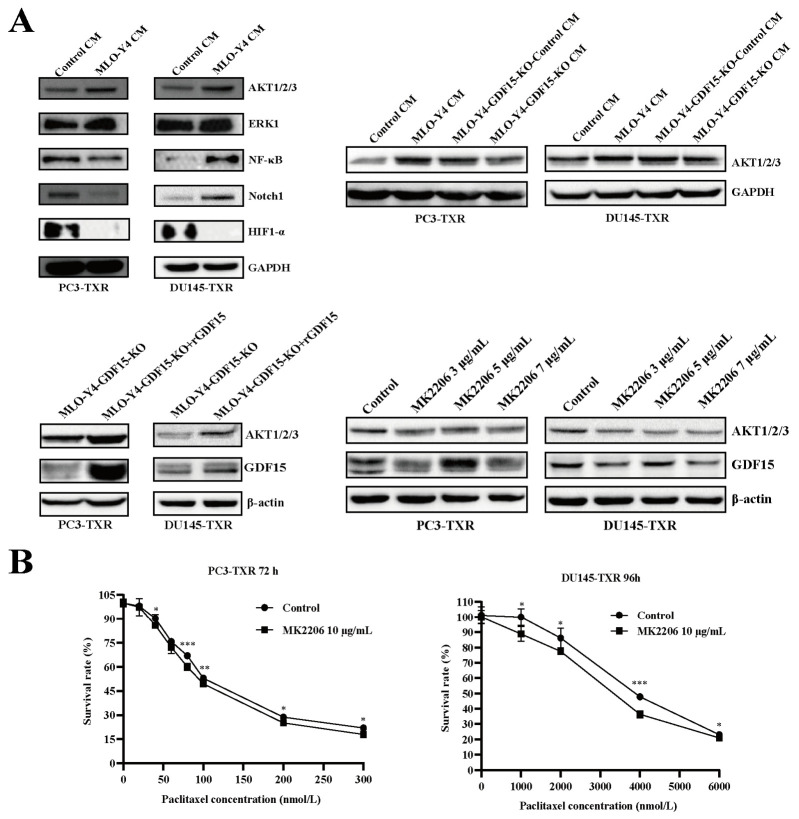
Western blotting analysis of transcription factor and signaling protein expression in paclitaxel-resistant prostate cancer cells under different conditioned medium and inhibitor treatments. (**A**) PC3-TXR and DU145-TXR cells were incubated with MLO-Y4 CM. Total cellular protein was extracted and subjected to Western blotting to detect the expression levels of AKT1/2/3, ERK1, NF-κB, Notch1, and HIF-1α. MLO-Y4-GDF15-KO CM was supplemented with rGDF15, and the prepared medium was used to treat PC3-TXR and DU145-TXR cells. Western blotting was performed to examine the expression changes in AKT1/2/3 and GDF15 in the two drug-resistant cell lines. PC3-TXR and DU145-TXR cells were treated with the AKT inhibitor MK2206. Cellular protein lysates were collected for Western blotting to detect the expression levels of AKT1/2/3 and GDF15. (**B**) PC3-TXR and DU145-TXR cells were treated with paclitaxel in the presence or absence of MK2206 (10 μg/mL). Data are shown as the mean ± SEM of three independent experiments. * *p* < 0.05, ** *p* < 0.01, *** *p* < 0.001.

To validate these findings, recombinant GDF15 (rGDF15) was added to conditioned medium from GDF15-knockout MLO-Y4 cells (MLO-Y4-GDF15-KO CM), and t rGDF15-supplemented CM was applied to drug-resistant prostate cancer cells. Addition of rGDF15 significantly restored AKT1/2/3 expression levels. To further explore the relationship between the AKT signaling pathway and GDF15-mediated paclitaxel resistance, PC3-TXR and DU145-TXR cells were treated with the AKT inhibitor MK2206 [38,39]. Inhibition of AKT signaling decreased GDF15 expression (Figure 4A) and concurrently reduced paclitaxel resistance (Figure 4B). CCK-8 assays revealed that the IC50 of PC3-TXR cells in the control group at 72 h was 138.97 ± 16.97 nmol/L, whereas treatment with 10 μg/mL MK2206 resulted in an IC50 of 119.47 ± 13.19 nmol/L. For DU145-TXR cells at 96 h, the IC50 was 3580.33 ± 582.11 nmol/L in the control group and 2828.33 ± 341.23 nmol/L following treatment with 10 μg/mL MK2206. These results demonstrate that GDF15 upregulates AKT1/2/3 expression in drug-resistant prostate cancer cells and that AKT signaling contributes to GDF15-mediated paclitaxel resistance.

### 3.5. GDF15 Induces EMT in Drug-Resistant Prostate Cancer Cells

**Figure 5:**
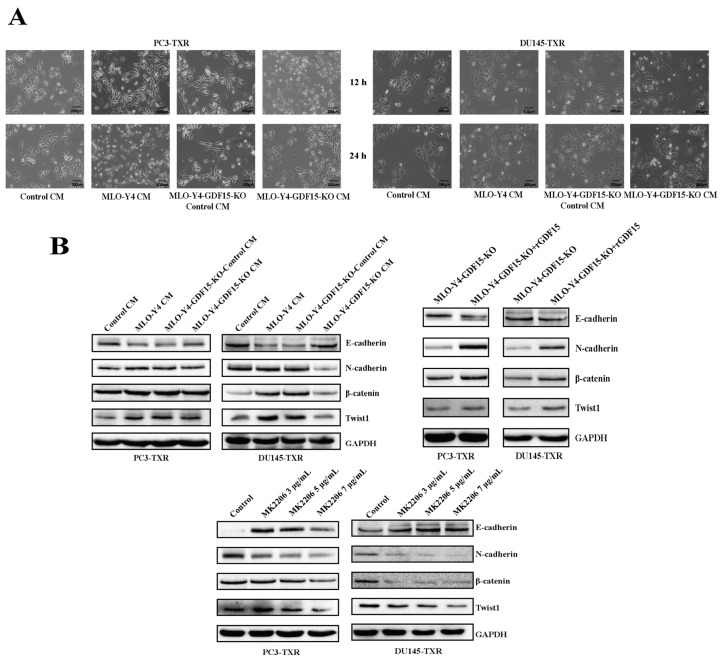
GDF15 induces EMT and promotes drug resistance in drug-resistant prostate cancer cells. (**A**) PC3-TXR and DU145-TXR cells were treated with control CM, MLO-Y4 CM, MLO-Y4-GDF15-KO-Control CM, or MLO-Y4-GDF15-KO CM for 24 h. Cell morphology was examined by light microscopy. (**B**) PC3-TXR and DU145-TXR cells were treated as in A, and total protein was subjected to Western blotting for E-cadherin, N-cadherin, β-catenin, and Twist1. To confirm GDF15-dependence, MLO-Y4-GDF15-KO CM was supplemented with rGDF15 cell treatment. To assess the involvement of AKT signaling, cells were treated with the AKT inhibitor MK2206.

After incubation of PC3-TXR and DU145-TXR cells with four distinct types of conditioned medium, significant changes in cell morphology were observed under a microscope (Figure 5A), wherein cells exhibited a spindle-shaped morphology and loose intercellular arrangement. We hypothesized that the cells may have undergone the epithelial–mesenchymal transition (EMT) process [40,41,42,43]. To confirm whether the cells had undergone EMT, the expression levels of EMT-related factors (E-cadherin, N-cadherin, β-catenin, and Twist1) after treatment with the four types of conditioned media were detected. The results showed that the expression level of E-cadherin decreased with an increase in GDF15 content in the microenvironment, whereas N-cadherin, β-catenin, and Twist1 displayed the opposite expression trend. To determine whether the EMT process was mediated by the microenvironmental GDF15, rGDF15 was supplemented into MLO-Y4-GDF15-KO CM for verification, and the results further confirmed the above conclusion. Additionally, after treating two drug-resistant prostate cancer cell lines with MK2206, the expression of EMT-related factors was detected, and the results were consistent with the above conclusions (Figure 5B). These results suggest that the content of GDF15 in the microenvironment is closely related to the activation of the AKT signaling pathway and the EMT process, and GDF15 may affect the paclitaxel resistance of prostate cancer drug-resistant cells by regulating the AKT signaling pathway and mediating the EMT process.

### 3.6. GDF15 Secreted by Bone Cells Promotes Prostate Tumor Growth In Vivo

**Figure 6:**
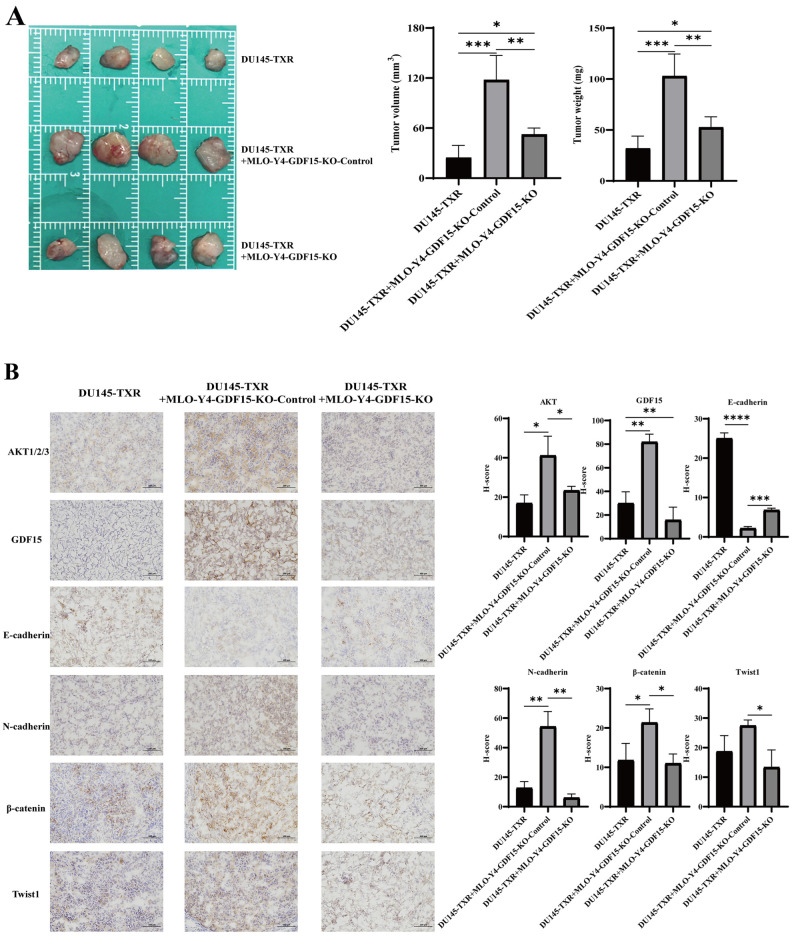
Osteocyte-derived GDF15 promotes DU145-TXR tumor growth and EMT in vivo. (**A**) DU145-TXR cells were injected subcutaneously alone or mixed with MLO-Y4-GDF15-KO-Control or MLO-Y4-GDF15-KO cells at a 10:1 ratio. Representative tumor images (left), tumor volumes (middle), and tumor weights (right) are shown. (**B**) Immunohistochemical analysis of E-cadherin, N-cadherin, β-catenin, Twist1, AKT1/2/3, and GDF15 in tumor tissues from each group. Representative images and quantification of staining are presented. Data are shown as mean ± SEM. * *p* < 0.05, ** *p* < 0.01, *** *p* < 0.001, **** *p* < 0.0001.

To investigate whether osteocyte-derived GDF15 facilitates the growth of drug-resistant prostate cancer cells in vivo, in this study, MLO-Y4-GDF15-KO-Control cells and MLO-Y4-GDF15-KO cells were co-mixed with DU145-TXR cells at a 10:1 ratio for subcutaneous injection into nude mice. Cell viability was assessed prior to injection to ensure viability exceeded 95%. The results showed that compared with the group that were injected with DU145-TXR cells alone, the group that were injected with MLO-Y4-GDF15-KO-Control cells and DU145-TXR cells together formed larger tumors, while the tumor volume in this co-injection group was markedly smaller than that in the control group (Figure 6A).

Immunohistochemical analysis was conducted on each group of tumor tissues. It was observed that the expression level of E-cadherin decreased as the concentration of GDF15 in the bone microenvironment increased. Meanwhile, the expression of AKT1/2/3, GDF15, N-cadherin, β-catenin, and Twist1 was elevated with increasing GDF15 abundance within the bone microenvironment (Figure 6B). These observations were consistent with our earlier in vitro data. These conclusions indicate that GDF15 in the bone microenvironment can induce higher expression of AKT1/2/3 and GDF15, as well as promote EMT progression in the bone microenvironment.

## 4. Discussion

Based on in vitro and in vivo experiments, our study elucidates the regulatory role and molecular mechanism by which osteocytes promote invasion, metastasis, and paclitaxel resistance in drug-resistant prostate cancer cells. Our core finding is the existence of a positive feedback loop between paclitaxel-resistant prostate cancer cells and osteocytes (MLO-Y4 cells): prostate cancer-resistant cells induce osteocytes to secrete GDF15. GDF15 expression is upregulated under pathological states including acute injury, chronic inflammation, and malignant tumors [21,23,44], and has been reported to promote osteoclast differentiation while inhibiting osteoblast differentiation [11,45]; however, its role in osteocytes has received little attention. Osteocytes are closely associated with tumor cell proliferation and metastasis [24,46,47,48], and emerging evidence suggests that GDF15 may promote drug resistance by activating multiple signaling pathways [12,49]. We demonstrate that osteocyte-derived GDF15 not only directly enhances the migration, invasion, and paclitaxel resistance of drug-resistant cells, but also upregulates GDF15 expression in the cancer cells themselves, an effect achieved through activation of the AKT signaling pathway and induction of EMT. These findings provide direct experimental evidence that the bone microenvironment (osteocytes) promotes drug resistance in prostate cancer cells, implicating local tumor–stroma interactions in the development of resistance [50,51]. The bone microenvironment is critical for prostate cancer metastasis and drug resistance. In this study, we used MLO-Y4 cells to model the bone microenvironment and focused on the role of osteocytes in paclitaxel resistance. While the roles of osteoblasts and osteoclasts in prostate cancer metastasis have been extensively investigated [14,15], the regulatory function of osteocytes remains poorly understood. Our targeted investigation of the interaction between osteocytes and drug-resistant prostate cancer cells thus offers a new perspective for advancing the mechanistic understanding of prostate cancer metastasis and drug resistance [52,53].

Our in vitro experiments confirm that drug-resistant prostate cancer cells can educate osteocytes through soluble factors, thereby enhancing their capacity to promote the malignant phenotype. In contrast to previous reports that tumor-derived pressure stimulates osteocytes to secrete CCL5, we identify GDF15 as the key effector molecule produced by osteocytes in response to drug-resistant prostate cancer cells. CRISPR/Cas9-mediated knockout of GDF15 in MLO-Y4 cells substantially attenuates the ability of their conditioned medium to promote the growth, invasion, and paclitaxel resistance of drug-resistant cells, directly confirming the central role of osteocyte-derived GDF15 in this interaction. These results establish that osteocytes exert a pro-tumorigenic function in paclitaxel-resistant prostate cancer: education of osteocytes by resistant cells through soluble factors significantly potentiates the ability of osteocytes to enhance tumor growth, invasion, and drug resistance, and this conclusion is supported by both our in vitro and in vivo data. Our data demonstrate that both osteocytes and prostate cancer cells can secrete GDF15. Specifically, Figure 2A reveals that conditioned medium derived from cancer cells markedly upregulates GDF15 expression in osteocytes above their basal levels. Meanwhile, Figure 2B illustrates that osteocyte-derived GDF15 further induces GDF15 production in taxane-resistant prostate cancer cells. Collectively, these results establish a bidirectional positive feedback loop between the two cell populations.

In the context of paclitaxel resistance, our study delineates the oncogenic function of GDF15. We found that GDF15 mRNA and protein levels were significantly elevated in drug-resistant prostate cancer cells compared with parental cells. MLO-Y4 osteocytes secreted high levels of GDF15, mirroring the elevated GDF15 levels observed in the serum of patients with prostate cancer metastasis [11,54], indicating that GDF15 may serve as a potential biomarker for metastasis and paclitaxel resistance and providing a clear therapeutic target. Furthermore, we confirmed that osteocyte-derived GDF15 significantly promotes proliferation, invasion, and paclitaxel resistance of drug-resistant prostate cancer cells, consistent with our findings that high GDF15 expression positively correlates with the resistant phenotype, further supporting the feasibility of targeting GDF15 for therapeutic intervention [55,56,57,58,59,60]. Previous research on parental prostate cancer cells established the GDF15–GFRAL–EGR1 signaling cascade as a core oncogenic pathway [26]. Nevertheless, our data do not support the involvement of GFRAL and EGR1 in taxane-resistant cells, and we have acknowledged this unvalidated regulatory axis as a key limitation of our study. Further mechanistic exploration of GDF15–GFRAL signaling in drug-resistant prostate cancer will be our primary priority in future investigations [61,62].

We identified the “GDF15–AKT–EMT” signaling axis as a key regulator of paclitaxel resistance and invasion in prostate cancer. In vitro, treatment with GDF15 or MLO-Y4 conditioned medium upregulated AKT1/2/3 expression and induced characteristic EMT changes (decreased E-cadherin, increased N-cadherin) in drug-resistant prostate cancer cells, alterations closely associated with chemoresistance [27,28]. The AKT inhibitor MK2206 effectively reversed these molecular changes and partially restored paclitaxel sensitivity, directly confirming the central role of this signaling axis in regulating drug resistance. In vivo, osteocytes promoted the growth of drug-resistant prostate cancer cells through GDF15 secretion. H&E staining revealed that tumors from the MLO-Y4-GDF15-KO-Control co-injection group exhibited densely arranged tumor cells with no evident sheet-like necrosis and vigorous proliferation, whereas tumors from the MLO-Y4-GDF15-KO group displayed multiple scattered necrotic foci and reduced proliferative activity, confirming that this tumor-promoting effect arises primarily from enhanced proliferation rather than inhibition of apoptosis. Recent studies confirm that mTOR and FOXO transcription factors act as core downstream mediators of AKT, mediating bone microenvironment-induced EMT and paclitaxel resistance in metastatic prostate cancer. A comprehensive dissection of the full GDF15-AKT-mTOR/FOXO regulatory network is beyond the scope of the present study and will be the major focus of our future investigations [63].

Taken together, our findings have three major implications. First, we establish a positive correlation between GDF15 expression and the paclitaxel-resistant, invasive phenotype of prostate cancer. Second, we reveal a GDF15-mediated positive feedback loop between drug-resistant prostate cancer cells and osteocytes. Third, we define the “GDF15–AKT–EMT” signaling axis, advancing the molecular understanding of prostate cancer chemoresistance. From a translational perspective, our results suggest three potential intervention strategies: targeting GDF15, inhibiting the downstream AKT pathway, and modulating the bone microenvironment, thus providing experimental support for overcoming chemoresistance in prostate cancer. In summary, our in vitro and in vivo studies demonstrate that drug-resistant prostate cancer cells and osteocytes engage in a GDF15-mediated positive feedback loop, and that the “GDF15–AKT–EMT” signaling axis plays a central role in paclitaxel resistance and metastasis. These findings highlight the importance of osteocytes in prostate cancer metastasis and provide direct experimental evidence and rationale for targeted therapy against chemotherapy resistance.

## Data Availability

All data included in this study are available upon request by contact with the corresponding author.
